# Stable transformation of *Babesia bigemina* and *Babesia bovis* using a single transfection plasmid

**DOI:** 10.1038/s41598-018-23010-4

**Published:** 2018-04-17

**Authors:** Marta G. Silva, Donald P. Knowles, Monica L. Mazuz, Brian M. Cooke, Carlos E. Suarez

**Affiliations:** 1 Department of Veterinary Microbiology and Pathology, Washington State University, Pullman, Washington, United States of America; 2 Animal Disease Research Unit, Agricultural Research Service, USDA, WSU, Pullman, Washington, United States of America; 3 Division of Parasitology, Kimron Veterinary Institute, P.O.B. 12, Bet Dagan, 50250 Israel; 4 Department of Microbiology, Biomedicine Discovery Institute, Monash University, Victoria, 3800 Australia

## Abstract

*Babesia bigemina* and *Babesia bovis*, are the two major causes of bovine babesiosis, a global neglected disease in need of improved methods of control. Here, we describe a shared method for the stable transfection of these two parasites using electroporation and blasticidin/blasticidin deaminase as a selectable marker. Stably transfected *B*. *bigemina* and *B*. *bovis* were obtained using a common transfection plasmid targeting the enhanced *green fluorescent protein-BSD* (*egfp-bsd*) fusion gene into the *elongation factor-1α* (*ef-1α*) locus of *B*. *bigemina* and *B*. *bovis* under the control of the *B*. *bigemina ef-1α* promoter. Sequencing, Southern blotting, immunoblotting and immunofluorescence analysis of parasite-infected red blood cells, demonstrated that the *egfp-bsd* gene was expressed and stably integrated solely into the *ef-1α* locus of both, *B*. *bigemina* and *B*. *bovis*. Interestingly, heterologous *B*. *bigemina ef-1α* sequences were able to drive integration into the *B*. *bovis* genome by homologous recombination, and the stably integrated *B*. *bigemina ef-1α*-A promoter is fully functional in *B*. *bovis*. Collectively, the data provides a new tool for genetic analysis of these parasites, and suggests that the development of vaccine platform delivery systems based on transfected *B*. *bovis* and *B*. *bigemina* parasites using homologous and heterologous promoters is feasible.

## Introduction

Bovine babesiosis caused by *Babesia bovis* and *B*. *bigemina* is an acute and persistent tick-borne disease with a high negative economic impact worldwide. The disease is characterized by high mortality and morbidity in susceptible animals that develop fever, anemia, jaundice, weight lost, reduction in milk production and, in severe cases, death. Animals that survive an acute infection become long-term asymptomatic carriers of the parasites. Persistently infected animals are therefore a continuous source for transmission by ticks and the maintenance of herd immunity^1^.

*B*. *bigemina* is usually regarded as having relatively reduced virulence compared to *B*. *bovis* however, *B*. *bigemina* is also responsible for important economic losses worldwide and improved methods of control are urgently needed. Currently control of babesiosis is achieved by acaricides and live-attenuated vaccines, but these approaches have serious limitations, including the development of acaricide resistance by ticks, and current efforts are focused on the development of novel and more effective recombinant protein sub-unit vaccines or genetically-attenuated parasites. The development of stable transfection systems for *B*. *bovis*^2,3^, have already facilitated several new avenues of research, including functional gene characterization^4^ and novel vaccine development^5,6^, but such methods are not available for *B*. *bigemina*. The development of genetic manipulation tools for *B*. *bigemina* will advance our understanding of parasite biology, gene function and improved control of bovine babesiosis^6–9^. A recent study described an effective *B*. *bigemina* promoter, interspecies (*B*. *bovis* and *B*. *bigemina*) activity for the elongation factor (*ef*)*−1α* promoters, and a method for incorporating exogenous DNA into *B*. *bigemina*, but appropriate selectable markers and a permissible site for stable integration of transfected genes remained undefined. However, initial *B*. *bovis* transfection systems were based on the use of the blasticidin and blasticidin deaminase (BSD) as a selectable marker, and the *ef-1α* locus as a permissible site for targeting exogenous gene integration^2^. Importantly, the availability of a partial *B*. *bigemina* genome (http://www.sanger.ac.uk/resources/downloads/protozoa/babesia-bigemina.html) and the previous characterization of the *B*. *bigemina ef-1α* locus^10^, indicate that the structure of this locus is essentially identical in both *B*. *bigemina* and *B*. *bovis*. Together, these observations suggests that it would be feasible to use a common strategy for gene integration in both *B*. *bovis* and *B*. *bigemina*, based on targeting one of the two identical *ef-1α* open reading frames (orfs) present in the *ef-1α* locus.

Here, we describe for the first time a stable transfection system for *B*. *bigemina* based on integration of the *egfp-bsd* gene under the control of the *ef-1α* promoter, into the *ef-1α* locus of *B*. *bigemina*, after drug selection with blasticidin. In addition, the same plasmid used for transfection of *B*. *bigemina* was able to insert and express foreign sequences in the *ef-1α* locus of *B*. *bovis*, thus expanding the options available for the genetic manipulation of *Babesia* sp. parasites more generally.

## Materials and Methods

### Ethics statement

Animals (*Bos taurus* Holstein steers, 12–20 months old) were used as blood donors for the maintenance of *in vitro* cultures of *B*. *bigemina* and *B*. *bovis* and were approved by the Institutional Animal Care and Use Committee (protocol 2013–66). All experiments performed in this study were conducted in accordance with the Protocol of Animal Usage Number 2013–66 approved by the University of Idaho IACUC Committee.

### *Invitro* parasite culture

*B*. *bigemina* (Puerto Rico strain)^11^ and *B*. *bovis* (T_3_Bo strain)^12^ were propagated in continuous microaerophilic stationary-phase culture as previously described. Briefly, *B*. *bigemina* and *B*. *bovis* cultures were grown in 96-well plates in bovine red blood cells (RBC) at 5% or 10% hematocrit, respectively, using HL-1 culture media at pH 7.2, and were incubated at 37 °C in an atmosphere of 5% CO_2_ and 90% N_2_^10^.

### Evaluation of sensitivity of *B*. *bigemina* and *B*. *bovis* to blasticidin

*B*. *bigemina* and *B*. *bovis* parasites were cultured in 180 µl of culture medium containing 5% and 10% bovine RBC, respectively, in a 96-well plate with different concentrations of blasticidin: 1, 1.2, 1.5, 1.8, 2, 4, 6, 8, 10, 12 and 14 μg/μl. Media without blasticidin was used as a control. The initial parasitemia was 0.2%. One hundred and fifty µl of culture media was daily replaced with corresponding amount of blasticidin. Percentage of parasitized erythrocytes (PPE) was monitored daily by Diff-Quik stained smears every 24 hr over a period of 72 hr by light microscopy (1000 × magnification). This experiment was carried out in triplicate.

### Parasite DNA extraction

Genomic DNA (gDNA) and plasmid DNA (pDNA) was extracted from cultured *B*. *bigemina* and *B*. *bovis* using the Qiagen Blood core kit according to the manufacturer’s instructions.

### Plasmid constructs

The 3′ and 5′ insertion target regions for stable transfection in the *ef-1-alpha-B* orf were generated by PCR with *B*. *bigemina* gDNA isolated from tissue culture cells utilizing the following sets of primers: Bbig-EF-orf-B-3′-*Bam*HI-F and Bbig-EF-orf-B-3′-*Bam*HI-R primers for the 3′ region of *B*. *bigemina ef* (expected size of 678 base pair (bp) amplicon]; Bbig-EF-orf-B-5′-Xho-F and Bbig-EF-orf-B-5′-Xho-R for the 5′ region of *B*. *bigemina ef* (expected size of 674 bp amplicon). These PCR products were cloned into the TOPO-TA 2.1 (Life technologies) cloning vector for sequence confirmation. The 3′ and 5′ insertion regions were then digested from the cloning vectors respectively with *Bam*HI and *Xho*I restriction enzyme and the amplicons were isolated and recovered from a 1% agarose gel. The plasmid containing the *ef-1α-A* promoter and luciferase gene along with the 3′ rap-1a stop region used in the *B*. *bigemina* transient transfection^10^ was digested with *Eco*RI restriction enzyme to remove the luciferase gene and the resulting linearized vector now containing just the *B*. *bigemina ef-1a-A* promoter and *B*. *bigemina* 3′ rap-1a stop region was re-circularized by ligation and transformed into TOP-10 *E*. *coli* competent cells (Life Technologies). The purified vector was then digested with *Bam*HI restriction enzyme and the 674 bp amplicon corresponding to 3′ ef-1*α*-B orf insertion region was ligated into this linear vector, transformed into TOP-10 cells and sequenced to confirm the correction orientation of the insertion. The resulting vector was digested with *Xho*I restriction enzyme and a similar procedure was used to ligate the 5′ ef-1*α*-B orf insertion target region into the vector. After confirming that both the 3′ and 5′ insertion sites were present in the correct orientation by sequencing, the vector was prepared for further ligation by digestion with *Eco*RI restriction enzyme. A synthetic *egfp-bsd* fusion gene from the p6-Cys-EKO plasmid (GenBank Accession number KX247384)^13^ containing *Eco*RI restriction sites was digested with *Eco*RI to remove the *egfp-bsd* fragment. This *egfp-bsd* fragment was isolated on a gel and the recovered fragment ligated into the vector containing the 3′ and 5′ *B*. *bigemina* insertion regions and the *rap-1a* 3′ stop region to generate the final stable transfection vector designated *pbig-ef-egfp-bsd*. The plasmid *pbig-ef-egfp-bsd* was used to transform TOP-10 cells, and plasmid was purified with the Qiagen Endotoxin free Plasmid Maxi Kit following the manufacturer’s instructions prior to transfection. The pBluescript (*pBS*) plasmid was used as a negative control in the transfection experiments.

### Transfection of *B*. *bigemina*

The transfection of *B*. *bigemina*-infected RBC was performed as described by Suarez *et al*.^2^. Briefly, *B*. *bigemina* iRBC with PPE ~20% was centrifuged at 600 x*g* for 5 min, and the cells washed once with 1 ml of Cytomix buffer (120 mM KCl, 0.15 mM CaCl_2_, 10 mM K_2_HPO_4_/KH_2_PO_4_ pH 7.6). Twenty μg of *pbig-ef-egfp-bsd* plasmid in 55 μl of cytomix was then gently mixed with 40 μl of washed *B*. *bigemina* iRBC then transferred to 0.2 cm electroporation cuvette and transfected by electroporation using a BioRad Gene Pulser II system at 1.2 kV + 25 µF + 200 Ω. After transfection, cells were immediately transferred to a 24-well plate containing 1.2 ml of HL-1 media with 5% of bovine RBC and 8 μg/μl of bsd to select for egfp-bsd expressing transgenic parasites.

### Transfection of *B*. *bovis*

The process for transfection of *B*. *bovis* parasites using plasmid *pbig-ef-egfp-bsd* was performed as described above for *B*. *bigemina*. After transfection, cells were immediately transferred to a 24-well plate containing 1.2 ml of HL-1 media with 10% of bovine RBC and 3 μg/μl of bsd to select for egfp-bsd expressing transgenic parasites.

### Confirmation of stable integration

#### Fluorescence microscopy

Two µl of either *B*. *bigemina-* (bigemina-big-ef-egfp-bsd) or *B*. *bovis-* (bovis-big-ef-egfp-bsd) transfected or wild-type cultures (non-transfected parasites) were observed by fluorescence microscopy (630 × magnification).

### Quantifying *invitro* growth of *B*. *bigemina* and *B*. *bovis*

*B. bigemina* (bigemina-big-ef-egfp-bsd) and *B*. *bigemina* wild-type parasites were cultured in 180 µl of culture medium containing 5% bovine RBC, in the absence of blasticidin and in the presence of 8 μg/μl of blasticidin. *B*. *bovis* (bovis-big-ef-egfp-bsd) and *B*. *bovis* wild-type parasites were cultured in 180 µl of culture medium containing 10% bovine RBC in the presence of 3 μg/μl of blasticidin or in the absence of blasticidin (positive control). The initial parasitemia was 0.2% and parasites were cultured in triplicate in 96-well plates. Medium (150 µl) was replaced daily. PPE was monitored every 24 hr up to 72 hr by Diff-Quik-stained RBC smears by optical microscope under 1000 × amplification. This experiment was carried out in triplicate.

### Southern blot analysis

Genomic DNA was extracted from wild-type *B*. *bigemina* and *B*. *bovis* parasites, *bigemina-big-ef-egfp-bsd* and *bovis-big-ef-egfp-bsd* with linearized *pBS* plasmid. One and half µg of gDNA were digested overnight with 10 units/µl of *BgI*II restriction enzyme and electrophoresis was carried out on a 1% agarose gel containing SYBR green dye. Three DIG-labeled were used: *B*. *bigemina rap-1* gene, a *B*. *bovis msa-1* gene, an *egfp-bsd* containing complete orf of *egfp* gene, a *B*. *bigemina ef-1α* gene (fwd 5′- CGCCTTTAGGGGCTTTACAACTCTGC-3′, rev 5′-CATTCCTGCTTAACGATACAGGC-3′) and a *B*. *bovis ef-1α* gene (fwd 5′-TGACATCTTTGAGAAATCTTAATGC-3′, rev 5′-ATCAGACAGATGTCTACTGAACTCGA GC-3′). Probes were DIG-labeled using a PCR DIG Probe Synthesis Kit (Roche) and hybridized with Dig Easy Hyb solution (Roche). Hybridizations of the DIG-labeled probes where detected using anti-digoxygenin Fab alkaline phosphatase conjugated (Roche) diluted 1/10.000 in blocking buffer, as described previously^2^. Roche Dig labeled DNA marker II was used as standard molecular marker, *pBS* control plasmid without *Babesia* promoter (*pBS* promoterless control plasmid) was used as plasmid control (Pr-) and *big-ef-egfp-bsd* plasmid control was used as a negative control.

### PCR amplification and sequencing

Sets of primers were designed to confirm the integration of plasmid *bigemina-big-ef-egfp-bsd* and *bovis-big-ef-egfp-bsd* by specific amplification of a DNA fragments surrounding the 5′ recombination site, the 3′ recombination site and the locus (Table 1). Amplicons were cloned into pCR™2.1-TOPO vector (Life Technologies) according to the manufacturer’s instructions and nucleotide sequences were confirmed by Sanger sequencing (ABI 3730).

**Table 1:** List of primers.

Name of the primer	Sequence 5′ to 3′
*egfp*-Fwd	ATG GTG AGC AAG GGC GAG
*ef1α*-Rev	TGT GAA ATA GGC TAG TGC CAG ATT CC
*egfp*-Rev	CTT GTA CAG CTC GTC CAT GC
*Bbov-Ups-efB*-Rev	GTT CCA TCA TGC TTC AGA GCA AAA AG
*rap-1a*-Fwd	CAC GAG GAA GGA ACT ACC GAT GTT GA
*rap-1a*-Rev	CCA AGG AGC TTC AAC GTA CGA GGT CA

### Immunoblot analysis

Proteins were extracted from cultured wild-type *B*. *bigemina* and *B*. *bovis*, bigemina-big-ef-egfp-bsd and bovis-big-ef-egfp-bsd and were used for immunoblot analysis as previously described^2^. Briefly, the immunoblots were incubated with mouse monoclonal antibody (MAb) against *B*. *bigemina* rap-1 protein (64/4.10.3)^14^ diluted to 2 µg/ml; *B*. *bovis* and bovis -big-ef-egfp-bsd were incubated with mouse MAb against *B*. *bovis* rap-1 protein (23/53.156.77) diluted to 2 µg/ml; wild-type *B*. *bigemina* and *B*. *bovis*, bigemina-big-ef-egfp-bsd and bovis-big-ef-egfp-bsd were incubated with rabbit anti-GFP diluted to 1:5,000. Membranes were subsequently incubated with HRP-conjugated goat anti-mouse IgG diluted 1:10,000 (on 64/4.10.3 and 23/53.156.77 antibody) or with HRP-conjugated goat anti-rabbit IgG diluted 1:5,000 (on anti-GFP antibody) for 45 minutes at RT. Chemiluminescent detection employed ECL™ western blotting substrate followed by exposure to X-film. Pre-immune mouse serum or non-infected bovine RBC were used as controls.

### Statistical analysis

Statistical significance was determined using ANOVA (GraphPad Prism 7 software). *P* < 0.05 were considered statistically significant.

## Results

### Growth inhibitory concentrations of blasticidin in *B*. *bigemina* and *B*. *bovis*

First we compared the inhibitory concentrations of blasticidin on *in vitro* cultured *B*. *bigemina* and *B*. *bovis*. Parasites were cultured in the presence of different concentrations of blasticidin ranging from 1 µg/µl to14 µg/µl, and the PPE was calculated daily up to 72 hr (Suppl. Figure 1a and b). The results show that increasing blasticidin concentrations above 4 μg/μl correlated with decreasing PPE for *B*. *bigemina*. The calculated IC_50_ found for *B*. *bigemina* and *B*. *bovis* was 3 µg/µl and 0.8 µg/µl, respectively. However 8 µg/µl and 3 µg/µl of blasticidin completely inhibited the growth of *B*. *bigemina* and *B*. *bovis* respectively (Suppl. Figure 1a and b), demonstrating that blasticidin as an appropriate selective inhibitory drug for developing a stable transfection system for *B*. *bigemina*.

### Generation of *B*. *bigemina* and *B*. *bovis* transfected lines stably expressing egfp-bsd under the control of a *B*. *bigemina ef-1α* promoter

**Figure 1:**
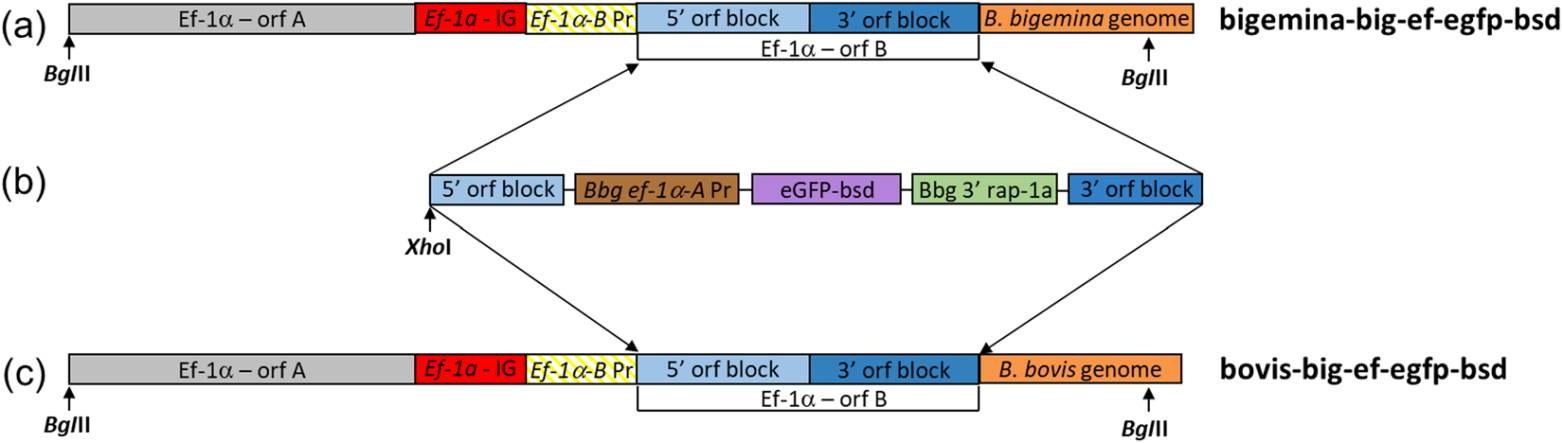
Experimental strategy used for the stable transfection of *B*. *bigemina* and *B*. *bovis* cell lines. (**a**) Schematic representation of the target *ef-1α* locus of *B*. *bigemina* in *B*. *bigemina* parasites (bigemina-big-ef-egfp-bsd). (**b**) Schematic representation of the stable transfection plasmid *pbig-ef-*e*gfp-bsd* used for electroporation. (**c**) Schematic representation of the target *ef-1α* locus of *B*. *bigemina* in *B*. *bovis* parasites (bovis-big-ef-egfp-bsd). *BgI*II: restriction enzyme.

**Figure 2:**
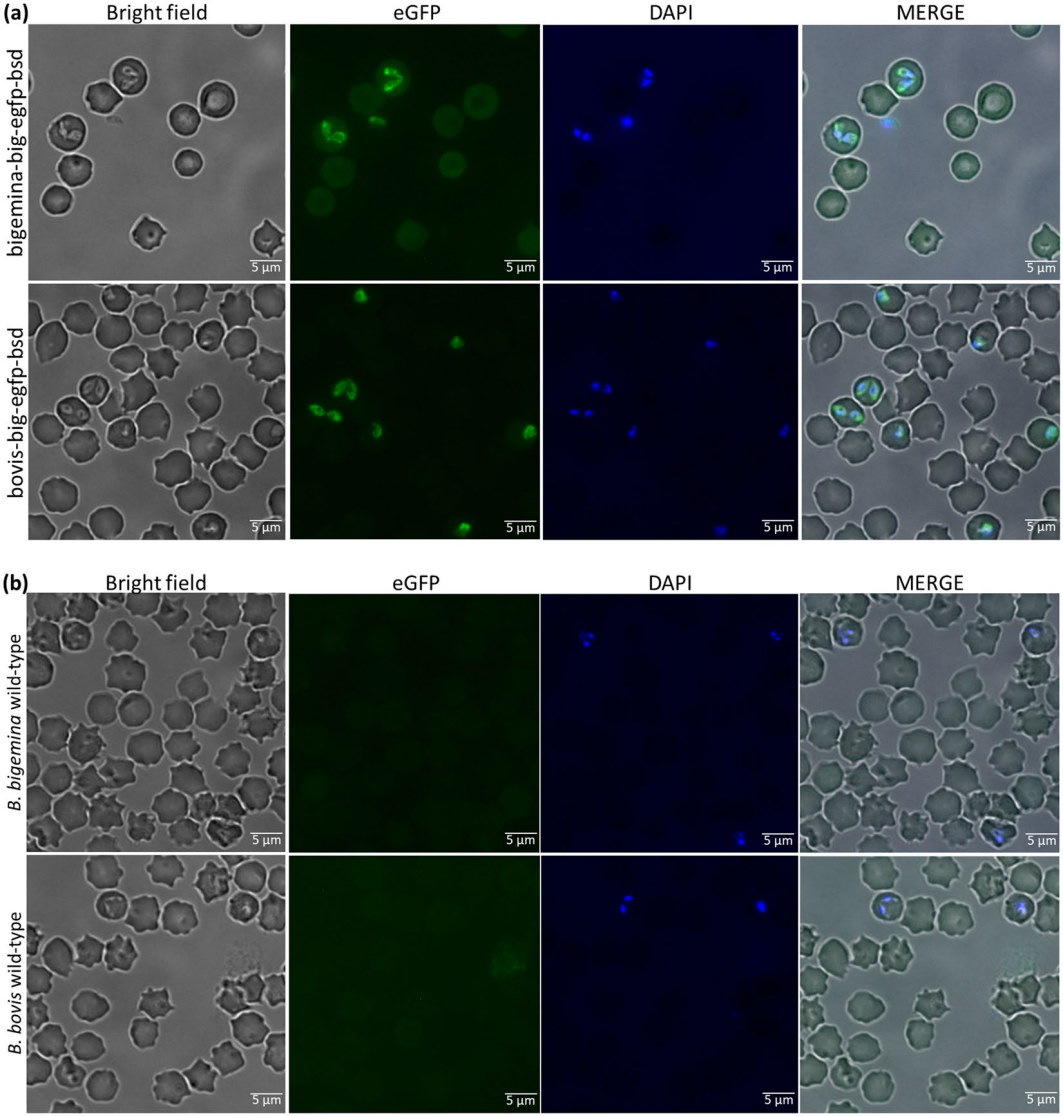
Analysis of *B*. *bigemina* and *B*. *bovis* parasites by fluorescence. (**a**) Bigemina-big-ef-egfp-bsd and bovis-big-ef-egfp-bsd transfected parasites observed under bright field, green laser (eGFP), blue laser (DAPI) and merge images. (**b**) *B*. *bigemina* and *B*. *bovis* wild-type parasites observed under bright field, green laser (eGFP), blue laser (DAPI) and merge images. Amplification 630x.

*B*. *bigemina* and *B*. *bovis* parasites were electroporated with plasmid *pbig-ef-*e*gfp-bsd* (Fig. 1a–c) followed by culturing in the presence of inhibitory doses of blasticidin. The transfection plasmid *pbig-ef-egfp-bsd* was designed for targeting the integration of the *egfp-bsd* selectable marker gene into the *ef-1α* locus of *B*. *bigemina* (Fig. 1b). Blasticidin resistant and green fluorescent *B*. *bigemina* and *B*. *bovis* parasites emerged 15 and 18 days after electroporation respectively, but no parasites were detectable in culture wells containing either control mock transfected or non-transfected or wild-type *B*. *bovis* and *B*. *bigemina* parasites growing in the presence of inhibitory doses of blasticidin (data not shown). The blasticidin-resistant parasites were maintained on blasticidin containing cultures for 2 months before analyzed for phenotypic and genotypic characterization. Analysis by fluorescence microscopy revealed intracellular expression of eGFP protein in the blasticidin-resistant *B*. *bigemina* and *B*. *bovis* transfected parasites, which were termed bigemina-big-ef-egfp-bsd and bovis-big-ef-egfp-bsd, respectively (Fig. 2a). As expected, no eGFP fluorescence was observed in *B*. *bigemina* and *B*. *bovis* wild-type control parasites (Fig. 2b).

### Phenotypic comparison between non-transfected and transfected *Babesia* parasites

**Figure 3:**
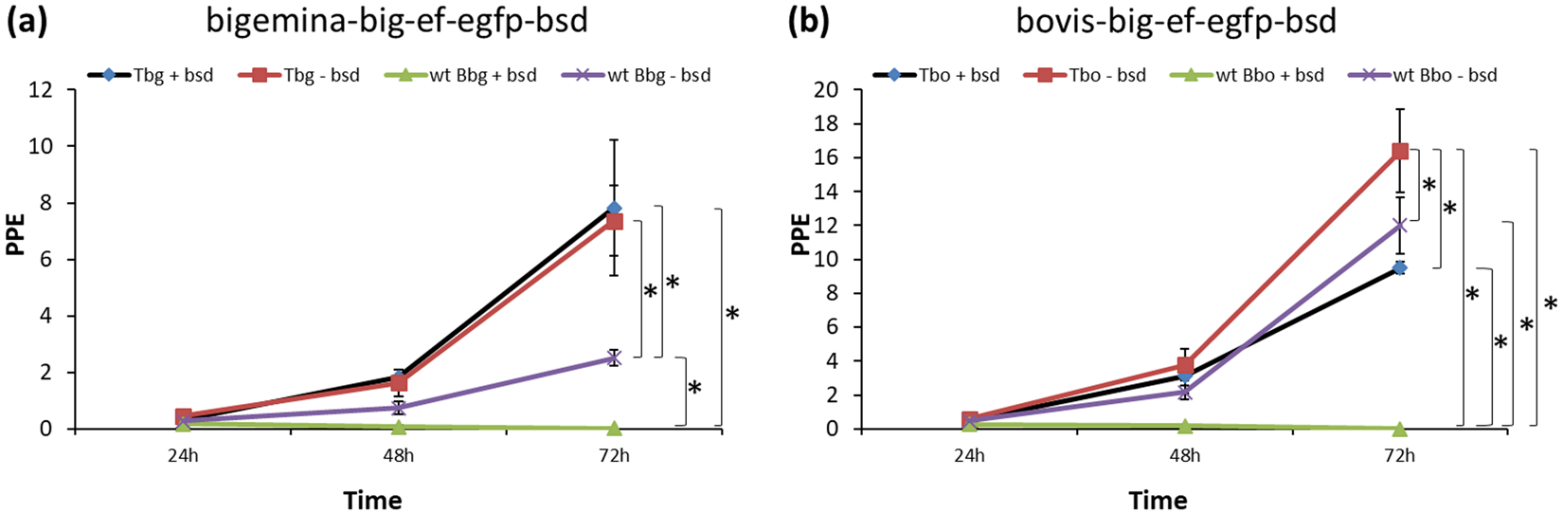
*In vitro* growth curve of *B*. *bigemina* and *B*. *bovis* parasites up to 72 hr. (**a**) *B*. *bigemina* growth curve. (**b**) *B*. *bovis* growth curve. Tbg + bsd: bigemina-big-ef-egfp-bsd parasites in the presence of blasticidin; Tbg-bsd: bigemina-big-ef-egfp-bsd parasites in the absence of blasticidin; wt Bbg + bsd: *B*. *bigemina* wild-type parasites in the presence of blasticidin; wt Bbg-bsd: *B*. *bigemina* wild-type parasites in the absence of blasticidin; Tbo + bsd: bovis-big-ef-egfp-bsd parasites in the presence of blasticidin; Tbo-bsd: bovis-big-ef-egfp-bsd parasites in the absence of blasticidin; wt Bbo + bsd: *B*. *bovis* wild-type parasites in the presence of blasticidin; wt Bbo-bsd: *B*. *bovis* wild-type parasites in the absence of blasticidin. Normalized PPE values (Y axis) obtained from *Babesia* spp. in the presence or absence of blasticidin (X axis). Media was used as negative controls for no inhibition. Error bars indicate standard deviations for each sample tested from triplicate culture. Data were compared using ANOVA analysis. *Represents *P* < 0.05 indicating a statistically significant difference between groups.

The *in vitro* growth rate of bigemina-big-ef-egfp-bsd and bovis-big-ef-egfp-bsd parasite lines, and wild-type parasites were compared. Notably, bigemina-big-ef-egfp-bsd parasites grew three times faster than wild-type parasites (*P* < 0.05) while bigemina-big-ef-egfp-bsd parasites grew at similar rate regardless of the presence or absence of blasticidin (Fig. 3a). However, as expected, *B*. *bigemina* wild-type parasites did not grow in the presence of blasticidin (Fig. 3a).

The bovis-big-ef-egfp-bsd parasites showed a higher rate of growth (*P* < 0.05) in blasticidin-free culture media (Fig. 3b) when compared to growth of bovis-big-ef-egfp-bsd parasites in the presence of blasticidin or the growth of wild-type parasites. Growth rates for big-ef-egfp-bsd and wild-type parasites in the presence or absence of blasticidin were indistinguishable (Fig. 3b). *B*. *bovis* wild-type parasites did not grow in the presence of blasticidin (Fig. 3b).

### Genotypic and proteomic characterization of stable *B*. *bigemina* and *B*. *bovis* transfected parasites

**Figure 4:**
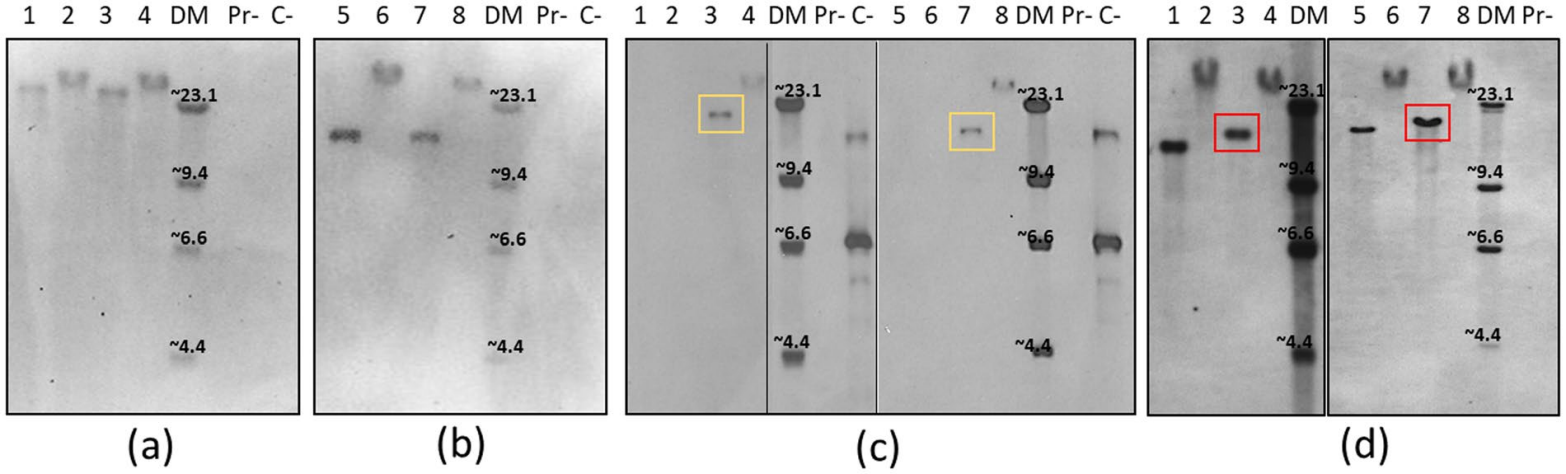
Southern blot analysis using dig-labeled probes against: (**a**) *B*. *bigemina rap-1a*; (**b**) *B*. *bovis msa-1*; (**c**) *egfp* and (**d**) *ef-1α*. 1: wild-type *B*. *bigemina* gDNA digested with *BgI*II; 2: wild-type *B*. *bigemina* gDNA undigested; 3: *bigemina-big-ef-egfp-bsd* digested with *BgI*II; 4: *bigemina-big-ef-egfp-bsd* undigested; DM: Dig labeled DNA marker II; Pr-: pBS promoterless control plasmid; C-: *pbig-ef-egfp-bsd* plasmid control; 5: wild-type *B*. *bovis* gDNA digested with *BgI*II; 6: wild-type *B*. *bovis* gDNA undigested; 7: *bovis-big-ef-egfp-bsd* digested with *BgI*II; 8: *bovis-big-ef-egfp-bsd* undigested. Vertical black stripes on the blot indicate cropping of the blot. A full image of the original blot can be seen in Supplementary Info section.

The genotypic characterization of transfected parasites was performed by Southern blot analysis, PCR and sequencing of PCR products. Genomic DNA from transfected bigemina-big-gfp-bsd and bovis-big-gfp-bsd cell lines, non-transfected *B*. *bovis* and *B*. *bigemina* parental strains, and plasmid *pbig-ef-egfp-bsd*, were analyzed in Southern blots using *B*. *bovis msa-1* (Fig. 4a), *B*. *bigemina rap-1* (Fig. 4b), *egfp* (Fig. 4c) and *ef-1α* (Fig. 4d) specific dig-labeled probes. The presence of a single band hybridizing with *egfp-bsd* probe, only in the transfected parasites is consistent with a single site integration of the exogenous transfected *egfp-bsd* gene in both bigemina-big-ef-egfp-bsd and bovis-big-ef-egfp-bsd cell lines (green boxes, Fig. 4c). Probing the blots with an *ef-1α* specific probe that hybridizes with sequences that are not included in the transfection constructs reveal an increase in the size of the *ef-1α* locus in the transfected parasites as a result of the insertion of the transfected genes. The calculated size of the *BgI*II restriction size containing the *ef-1α* locus of wild type *B*. *bigemina* and *B*. *bovis* are 16.4 and 18.8 Kb, respectively. However, the size of the locus, as detected by the specific labeled probe, was increased to 18.6 and 21 Kb, respectively, in both transfected parasite lines which matches with the predicted size of the stably inserted DNA (2.2 kb). As expected, the *rap-1* and *msa-1* probes react with identical patterns in transfected and non-transfected gDNA. Taken together, these results are consistent with stable integration of a single *egfp-bsd* gene copy in both parasite species.

**Figure 5:**
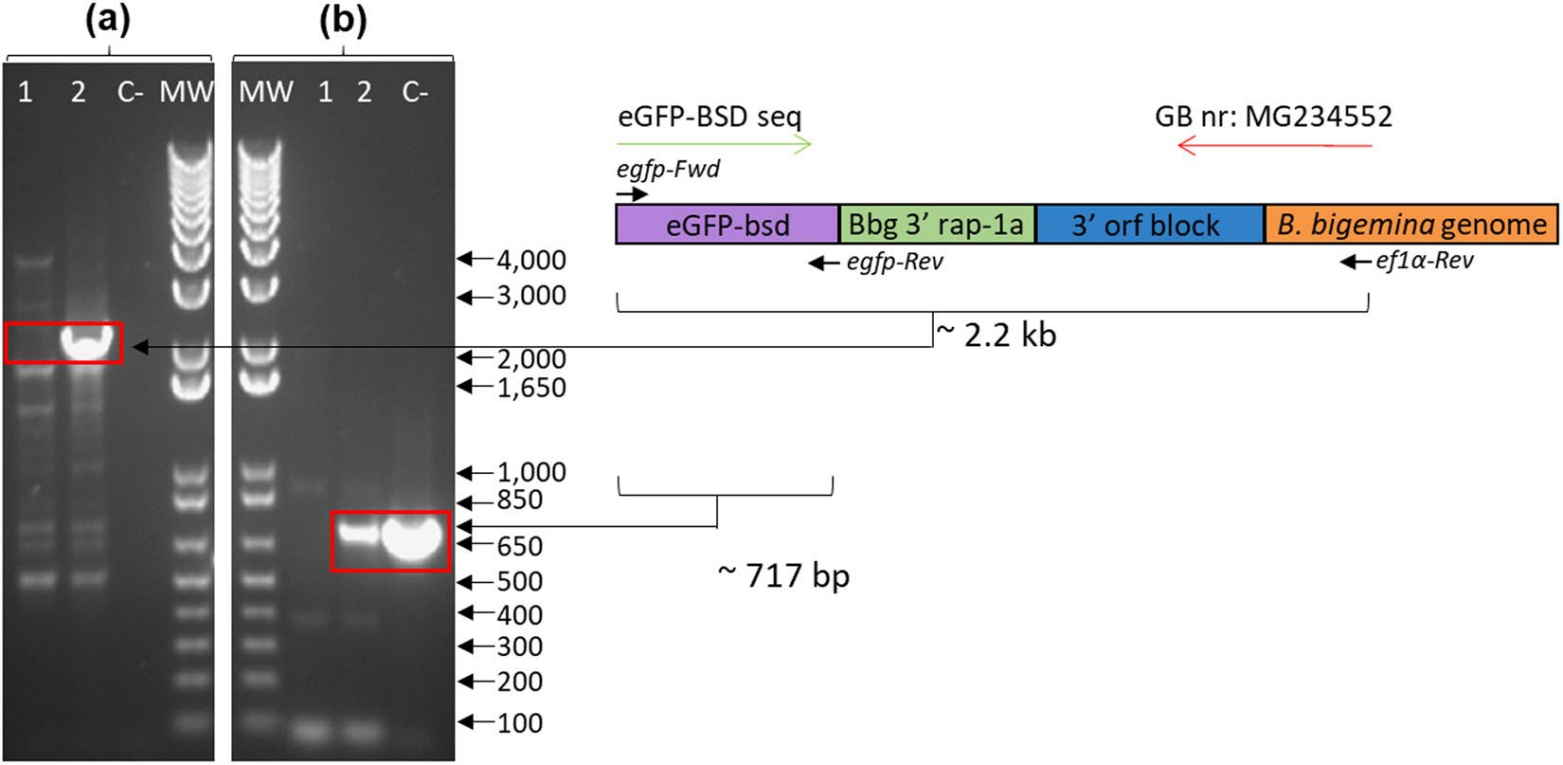
PCR integration analysis in *B*. *bigemina* using two different set of primers. (**a**) *egfp-Fwd* and *ef1α-*Rev. (**b**) *egfp*-Fwd and *egfp*-Rev. Line 1: wild-type *B*. *bigemina* gDNA; Line 2: *bigemina-big-ef-egfp-bsd*; C-: *pbig-ef-egfp-bsd* plasmid control; MW: molecular size ladder in bp, 1 Kb Plus DNA ladder.

**Figure 6:**
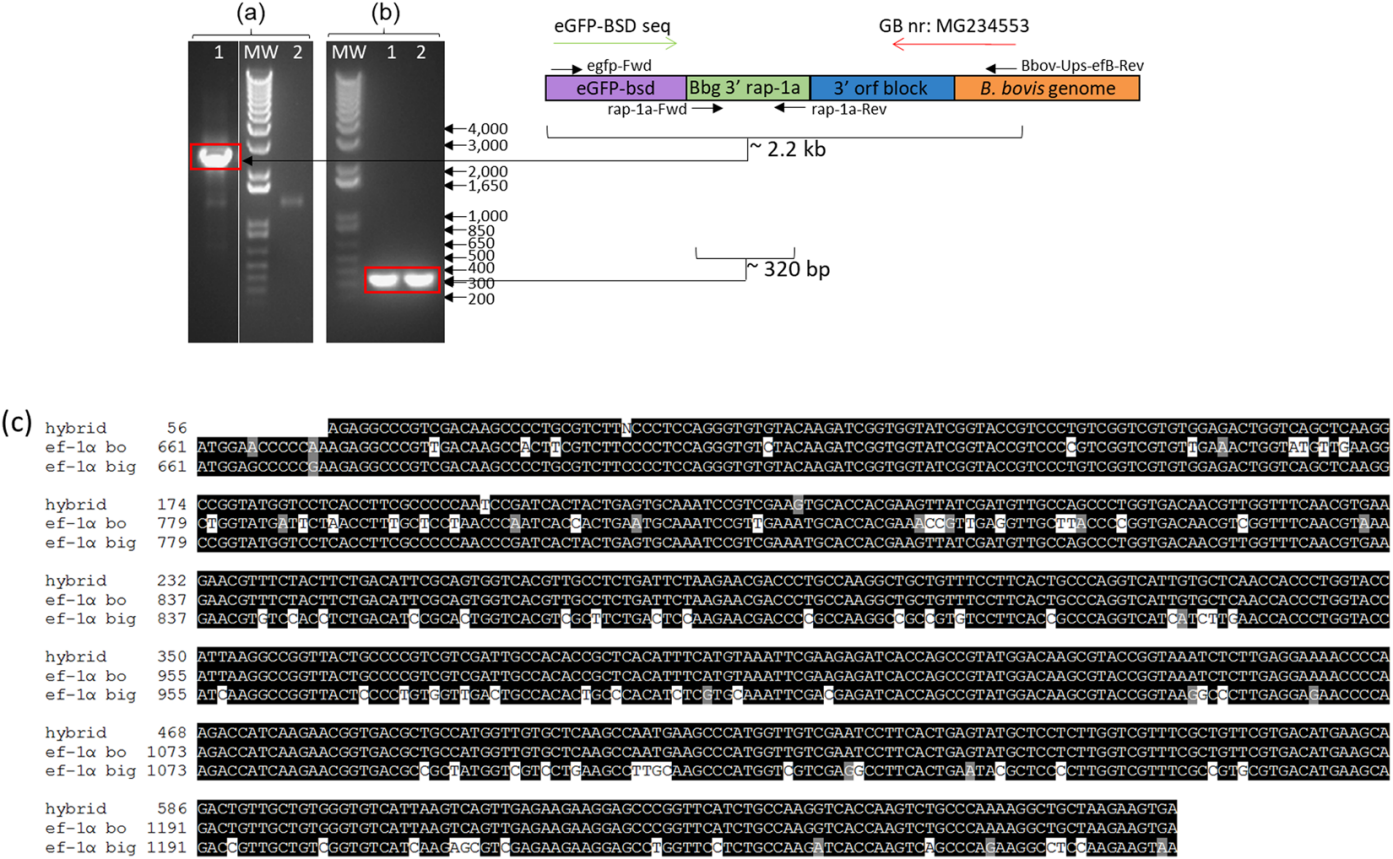
PCR integration analysis in *B*. *bovis* culture using two different sets of primers. (**a**) egfp-Fwd and Bbov-UpS-efB-Rev. (**b**) rap-1a-Fwd and rap-1a-Rev. Line 1: *bovis-big-ef-egfp-bsd* gDNA; line 2: wild-type gDNA *B*. *bovis*; MW: molecular size ladder in bp, 1 Kb Plus DNA ladder. (**c**) Alignments among the regions of insertion of the transfected genes into *bigemina-big-ef-egfp-bsd* and *bovis-big-ef-egfp-bsd* parasites. Vertical black stripes on the blot indicate where the image was cropped. Full image of the original agar electrophoresis gel can be seen in Supplementary Info section.

We then designed a PCR aimed at demonstrating correct integration of the exogenous gene into the *B*. *bigemina ef-1α* locus using primers based on the transfected *egfp* gene and sequences adjacent to the *ef-1α* locus of *B*. *bigemina* and *B*. *bovis* that are not present in the transfection plasmids (Figs 5 and 6, respectively). Genomic DNA derived from stably transfected and non-transfected (wild-type) control *B*. *bigemina* parasites were amplified by PCR using the set primers: A) egfp-Fwd and ef1α-Rev; and B) egfp-Fwd and egfp-Rev (Fig. 5 and Table 1). The expected band of 2.2 kb was observed only upon amplification of the transfected parasite line bigemina-big-ef-egfp-bsd but not on the gDNA derived from the wild-type *B*. *bigemina* parasites (Fig. 5a). The sequence of the 2.2 kb PCR amplicon was consistent with integration of the transfected *egfp-bsd* gene and its flanking regions into the B *ef-1α* gene of *B*. *bigemina* by homologous recombination (GenBank accession nr: MG234552). In addition, control PCR reactions using primers representing sequences present only in the transfection plasmid (Fig. 5b) (egfp-Fwd and egfp-Rev) only amplify a similar fragment in the transfected line bigemina-big-ef-egfp-bsd and transfection plasmid *pbig-ef-egfp-bsd*, but not on the gDNA derived from the wild-type *B*. *bigemina* parasites.

PCR amplifications performed using similar sets of primers on transfected bovis-big-ef-egfp-bsd and wild-type *B*. *bovis* parasites are shown in Fig. 6. Integration PCR using the set of primers egfp-Fwd and Bbov-UpS-efB-Rev yielded a ~2.2 kb band (Fig. 6a and Table 1) in gDNA of *bovis-big-ef-egfp-bsd* but not in gDNA from wild-type *B*. *bovis* parasites. Sequencing of the 2.2 kb amplicon is consistent with integration of the transfected *egfp-bsd* gene and its flanking regions into the B *ef-1α* gene of *B*. *bovis* by homologous recombination (GenBank accession nr: MG234553). Interestingly, sequencing of the PCR amplicon demonstrates the generation of a theoretically predictable hybrid partial *B*. *bigemina*-*B*. *bovis ef-1α* molecule in the transfected parasite. A comparison among the sequences of the hybrid portion of the molecule and the *ef-1α* DNA sequence of *B*. *bovis* and *B*. *bigemina* for the region involved in the insertion of the transfected genes into the genomes of the parasites is shown in Fig. 6b. Figure 6c also show alignments among the regions of insertion of the transfected genes into *B*. *bovis* and *B*. *bigemina* transfected parasites. PCR amplifications using primers rap-1a-Fwd and rap-1a-Rev confirmed the presence of the *rap-1a* gene in gDNA from transfected and wild-type parasites (Fig. 6b). An amplicon of ~320 bp was obtained (Fig. 6b).

**Figure 7:**
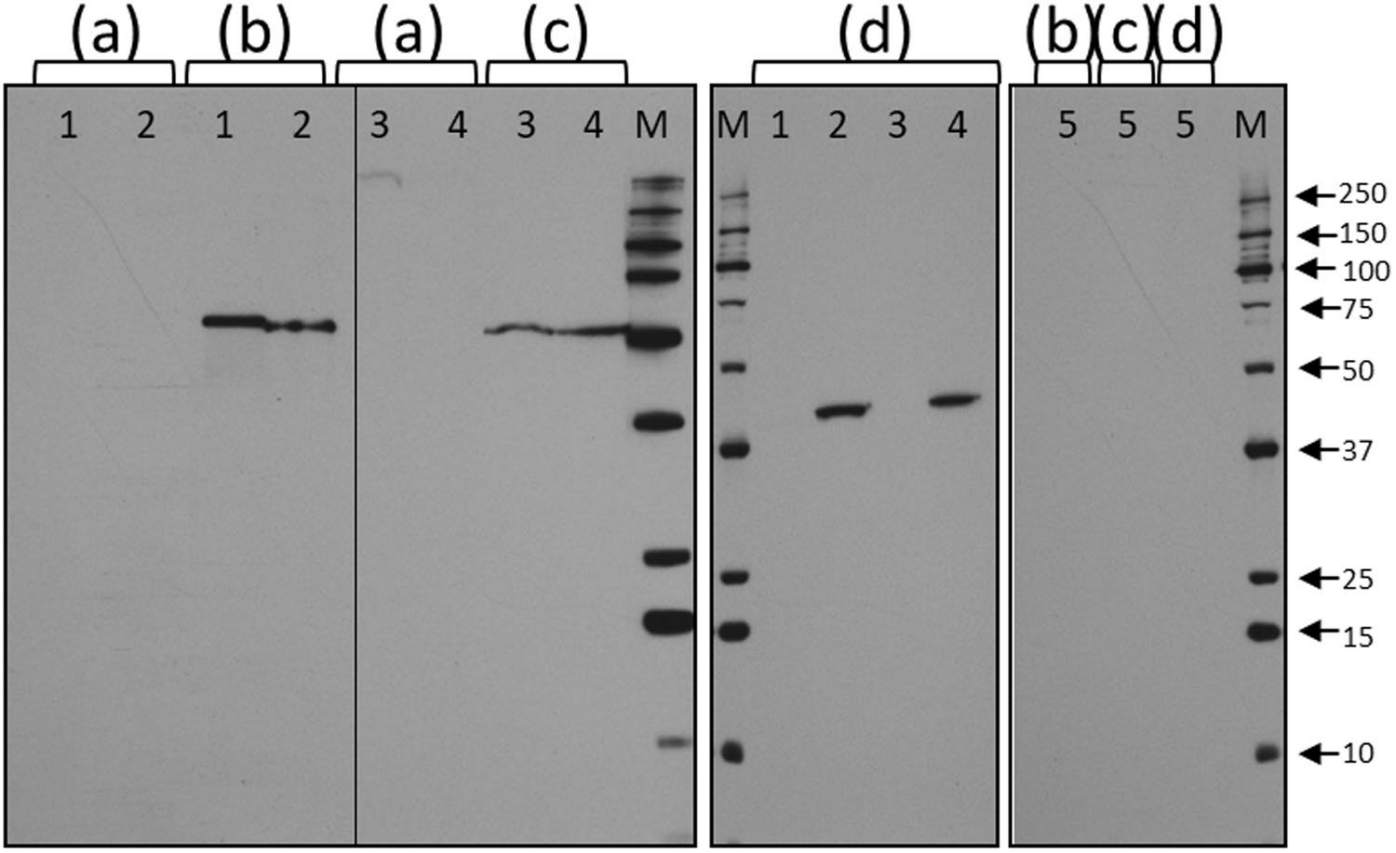
Immunoblot analysis. (**1**) wild-type *B*. *bigemina*. (**2**) bigemina-big-ef-egfp-bsd. (**3**) wild-type *B*. *bovis*. (**4**) bovis-big-ef-egfp-bsd. (**5**) Uninfected bovine RBC. Samples were incubated with antibodies: (**a**) pre-immune mouse serum; (**b**) anti-*B*. *bigemina* rap-1 MAb. (**c**) anti-*B*. *bovis* rap-1 MAb. (**d**) anti-GFP MAb. (M) molecular size ladder in kDa indicated by arrows. Vertical black stripes on the blot indicate cropping of the blot. A full image of the original blot can be seen in Supplementary Info section.

We also performed immunoblotting to confirm expression of eGFP-BSD by the transfected *B*. *bigemina* and *B*. *bovis* parasites. As can be seen in the immunoblot, no reactivity was observed for any of the antibodies used and uninfected bovine RBC (Fig. 7a and lanes 5). The wild-type *B*. *bigemina* and bigemina-big-ef-egfp-bsd reacted against *B*. *bigemina* rap-1 protein (~50 kDa) and the *B*. *bovis* and bovis-big-ef-egfp-bsd reacted against *B*. *bovis* rap-1 protein (~50 kDa) (Fig. 7b,c) using anti-RAP-1 MAbs as positive controls. Also, anti-GFP antibodies reacted with proteins present in both bigemina-big-ef-egfp-bsd and bovis-big-ef-egfp-bsd parasite lines with the expected molecular weight of the GFP-BSD fusion protein (~40 kDa), but not in wild-type *B*. *bigemina* and *B*. *bovis* (Fig. 7d).

Taken together, Southern blot, PCR and immunoblot data confirmed stable integration of the *egfp-bsd* gene into the genomes of both *B*. *bovis* and *B*. *bigemina* with demonstrated expression of the transfected exogenous genes.

## Discussion

Here, a stable transfection of *B*. *bigemina* and *B*. *bovis* using identical *B*. *bigemina* insertion and gene regulatory sequences that can be used for functional gene characterization or for the delivery of exogenous antigens by *Babesia* spp. parasites is described.

Importantly, the transfected *egfp-bsd* gene integrated as a single copy in the expected *ef-1α* locus in transfected *B*. *bigemina* parasites and no episomal forms of exogenous DNA bigemina-big-ef-gfp-bsd were detectable in parasites that were selected with blasticidin for at least two months.

A similar pattern of specific integration by means of homologous recombination for the exogenous transfected *egfp-bsd* gene into the expected *ef-1α* locus was also found to occur in *B*. *bovis* parasites using a plasmid transfection vector designed for integration into the *B*. *bigemina ef-1a* locus, despite the occurrence of sequence divergence in the *ef-1α* gene among the two species. Sequence comparisons among the ef-1*α* orf of *B*. *bovis* and *B*. *bigemina* show a level of identity of 87.45%. (Suppl. Figure 2 and Suppl. Table 1). Remarkably, that level of identity was sufficient to allow specific integration of the *pbig-ef-egfp-bsd* gene into the *ef-1α* locus of *B*. *bovis* genome, generating a hybrid *ef-1α* molecule in transfected *B*. *bovis*. The DNA sequence comparisons between *ef-1α* locus of *B*. *bigemina* and *B*. *bovis* (Suppl. Figure 2) provide hints on the mechanisms of homologous recombination operating in the parasite. Additionally, and consistent with previous findings^10^, the *ef-1α* promoter of *B*. *bigemina* was able to generate expression levels of the *egfp-bsd* gene that are sufficient to sustain growth of transfected parasites at high levels of blasticidin. The strategy for exogenous gene insertion used in this study makes expression of the transfected *egfp-bsd* gene by the “native” *B*. *bigemina* promoter theoretically possible, but this possibility is highly unlikely since the homologous or heterologous *ef-1α* promoter region (~700 bp), located immediately downstream the truncated ef-1α orf contain numerous stop codons. In addition, we previously demonstrated heterologous promoter function using transient transfection, where the transfected gene is expressed under the control of the sequences present in the transfection plasmid, and without the intervention of the original promoters, supporting the contention that the exogenous integrated promoter is indeed responsible for the expression of the *egfp-bsd* gene in the stably transfected parasites. Transfected *B*. *bigemina* parasites grew three times faster than non-transfected (wild-type) parasites. This might be due to changes in the regulation of the expression of *ef-1α* locus in the transfected parasites. It will be interesting to determine whether these changes affect the ability and efficiency of the parasite to infect bovine and tick hosts, and whether they are associated with parasite virulence.

The ability to transfect *B*. *bigemina* and *B*. *bovis* using *B*. *bigemina* insertion and promoter sequences also has important implications for improving the design of *Babesia* sp.-based vectored vaccines^6^. On one hand, a vaccine delivery platform based on *B*. *bigemina* transfected parasites might be more advantageous compared with *B*. *bovis* since the former parasite is known to cause less severe clinical disease in cattle, and, in contrast to *B*. *bovis*, does not result in microvascular sequestration of iRBC in the host. In contrast to *B*. *bovis*, *B*. *bigemina* parasites can be cleared from persistently infected animals, and dual *B*. *bovis*-*B*. *bigemina* infections are frequent in cattle in endemic areas^15–17^.

Possible future applications of this transfection platform include the use of attenuated *B*. *bigemina*-transfected parasites that express *B*. *bovis* and/or vector tick antigens that induce parasite and/or vector controlling immunity during subclinical persistent infection. These vectored vaccines might become ideal to generate protective immunity effective against both bovine parasites and their vector.

It will also be necessary to test the functionality of the *B*. *bigemina ef-1α* promoter in distinct life stages using transfected *B*. *bigemina* and *B*. *bovis* and parasites. These observations could have practical consequences for developing improved methods for the study and control of these parasites at different life-cycle stages.

In summary, we describe an efficient method for the stable transfection of *B*. *bigemina* that can be used for the future development of novel vaccines. These will require assuring that the transfected parasites are safe to deploy and that the genetic modifications do not result in undesirable phenotypic characteristics in potential vaccine candidate strains. The observation that the *B*. *bigemina-*specific construct can also be effectively and specifically used to transfect *B*. *bovis* parasites expands the range of possibilities toward the development of novel vaccines. Future work is aimed at determining whether transfected *B*. *bigemina* parasites are able to express exogenous or homologous antigens as vaccine platforms, and work as an effective method for functional genetic analysis in *B*. *bigemina*. The use of *B*. *bigemina* promoters in stably transfected *B*. *bovis* parasites also expands the toolbox available for the genetic manipulation of this parasite towards improved gene function characterization and vaccine development.

## Electronic supplementary material

Supplementary Information

Supplementary Dataset
