# Supplementary material for: Stable transformation of Babesia bigemina and Babesia bovis using a single transfection plasmid
Source: Sci Rep. 2018 Apr 17;8:6096. doi: 10.1038/s41598-018-23010-4 (PMC5904164; doi:10.1038/s41598-018-23010-4)
Supplement: Supplementary file 1 — Supplementary Information [file 41598_2018_23010_MOESM1_ESM.pdf]

Stable transformation of *Babesia bigemina* and *Babesia bovis* using a single transfection plasmid

Marta G. Silva[1*], Donald P. Knowles[1, 2], Monica L. Mazuz[3], Brian M. Cooke[4], and Carlos E. Suarez[1, 2]

[1]Department of Veterinary Microbiology and Pathology, Washington State University, Pullman, Washington, United States of America

[2]Animal Disease Research Unit, Agricultural Research Service, USDA, WSU, Pullman, Washington, United States of America

[3]Division of Parasitology, Kimron Veterinary Institute, P.O.B. 12, Bet Dagan, 50250 Israel

[4]Department of Microbiology, Biomedicine Discovery Institute, Monash University, Victoria 3800, Australia

*Corresponding author: Phone: +01-509-335-7321, Fax: +01-509-335-8328

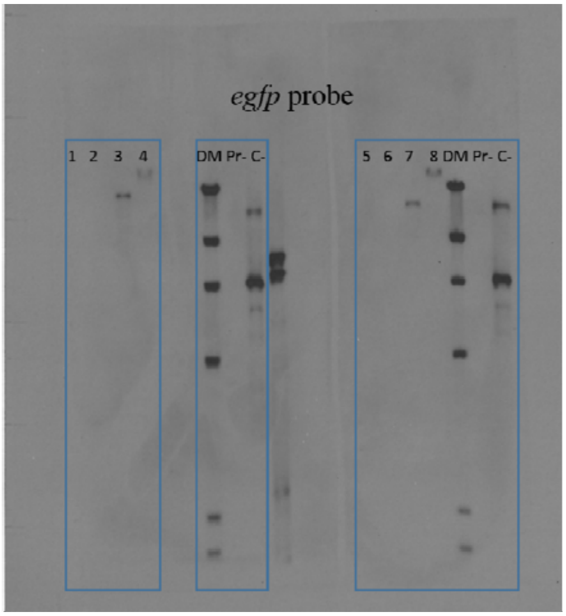

Supplementary Information Figure 4c. Southern blot analysis using dig-labeled *egfp* probes. **1**: wild-type *B. bigemina* gDNA digested with *Bgl*II; **2**: wild-type *B. bigemina* gDNA undigested; **3**: *bigemina-big-ef-egfp-bsd* digested with *Bgl*II; **4**: *bigemina-big-ef-egfp-bsd* undigested; DM: Dig labeled DNA marker II; Pr-: pBS promoterless control plasmid; C-: *pbig-ef-egfp-bsd* plasmid control; **5**: wild-type *B. bovis* gDNA digested with *Bgl*II; **6**: wild-type *B. bovis* gDNA undigested; **7**: *bovis-big-ef-egfp-bsd* digested with *Bgl*II; 8: *bovis-big-ef-egfp-bsd* undigested.

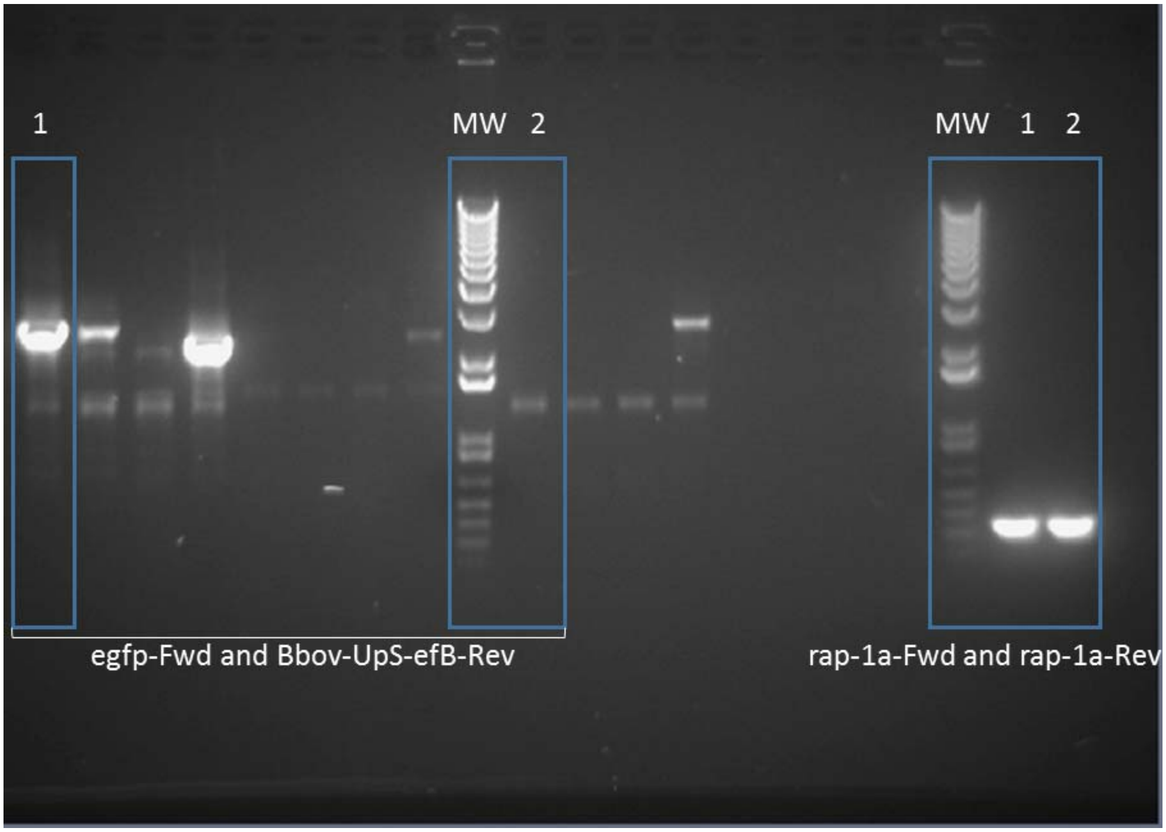

Supplementary Information Figure 6a and b. PCR integration analysis in *B. bovis* culture using two different sets of primers: egfp-Fwd and Bbov-UpS-efB-Rev; and rap-1a-Fwd and rap-1a-Rev. **Line 1**: *bovis-big-ef-egfp-bsd* gDNA; **line 2**: wild-type gDNA *B. bovis*; MW: molecular size ladder in bp, 1 Kb Plus DNA ladder

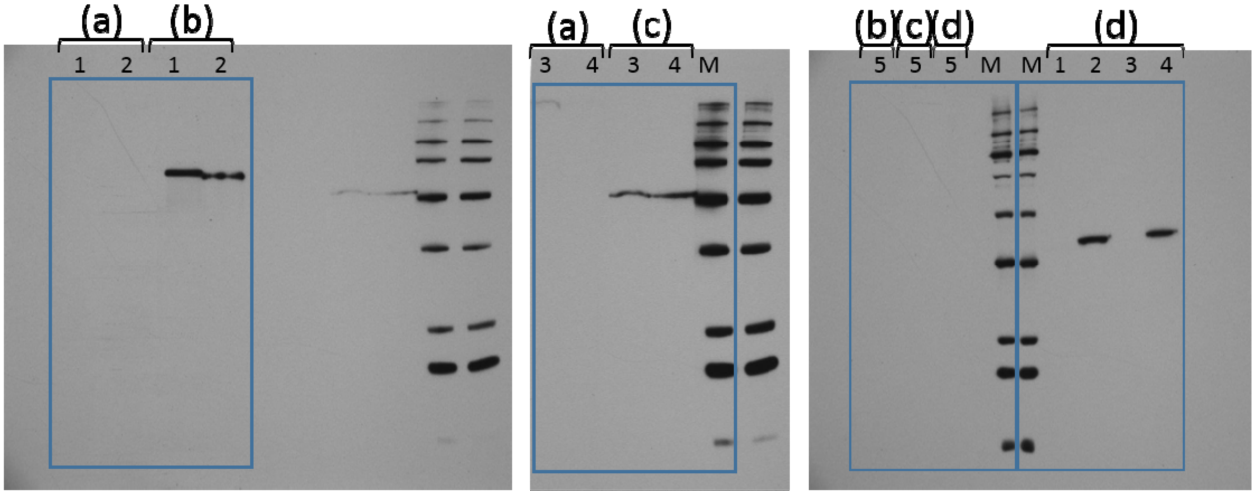

Supplementary Information Figure 7a, 7b and c. Immunoblot analysis. (**1**) wild-type *B. bigemina*. (**2**) bigemina-big-ef-egfp-bsd. (**3**) wild-type *B. bovis*. (**4**) bovis-big-ef-egfp-bsd. (**5**) Uninfected bovine RBC. Samples were incubated with antibodies: (**a**) pre-immune mouse serum; (**b**) anti-*B. bigemina* rap-1 MAb. (**c**) anti-*B. bovis* rap-1 MAb. (**d**) anti-GFP MAb. (M) molecular size ladder in kDa.
