# Supplementary material for: Stable transformation of Babesia bigemina and Babesia bovis using a single transfection plasmid
Source: Sci Rep. 2018 Apr 17;8:6096. doi: 10.1038/s41598-018-23010-4 (PMC5904164; doi:10.1038/s41598-018-23010-4)
Supplement: Supplementary file 2 — Supplementary Dataset [file 41598_2018_23010_MOESM2_ESM.docx]

**Stable transformation of *Babesia bigemina* and *Babesia bovis* using a single transfection plasmid**

Marta G. Silva^1*^, Donald P. Knowles^1, 2^, Monica L. Mazuz^3^, Brian M. Cooke^4^, and Carlos E. Suarez^1, 2^

^1^Department of Veterinary Microbiology and Pathology, Washington State University, Pullman, Washington, United States of America

^2^Animal Disease Research Unit, Agricultural Research Service, USDA, WSU, Pullman, Washington, United States of America

^3^Division of Parasitology, Kimron Veterinary Institute, P.O.B. 12, Bet Dagan, 50250 Israel

^4^Department of Microbiology, Biomedicine Discovery Institute, Monash University, Victoria 3800, Australia

***Corresponding author: Phone: +01-509-335-7321, Fax: +01-509-335-8328

**
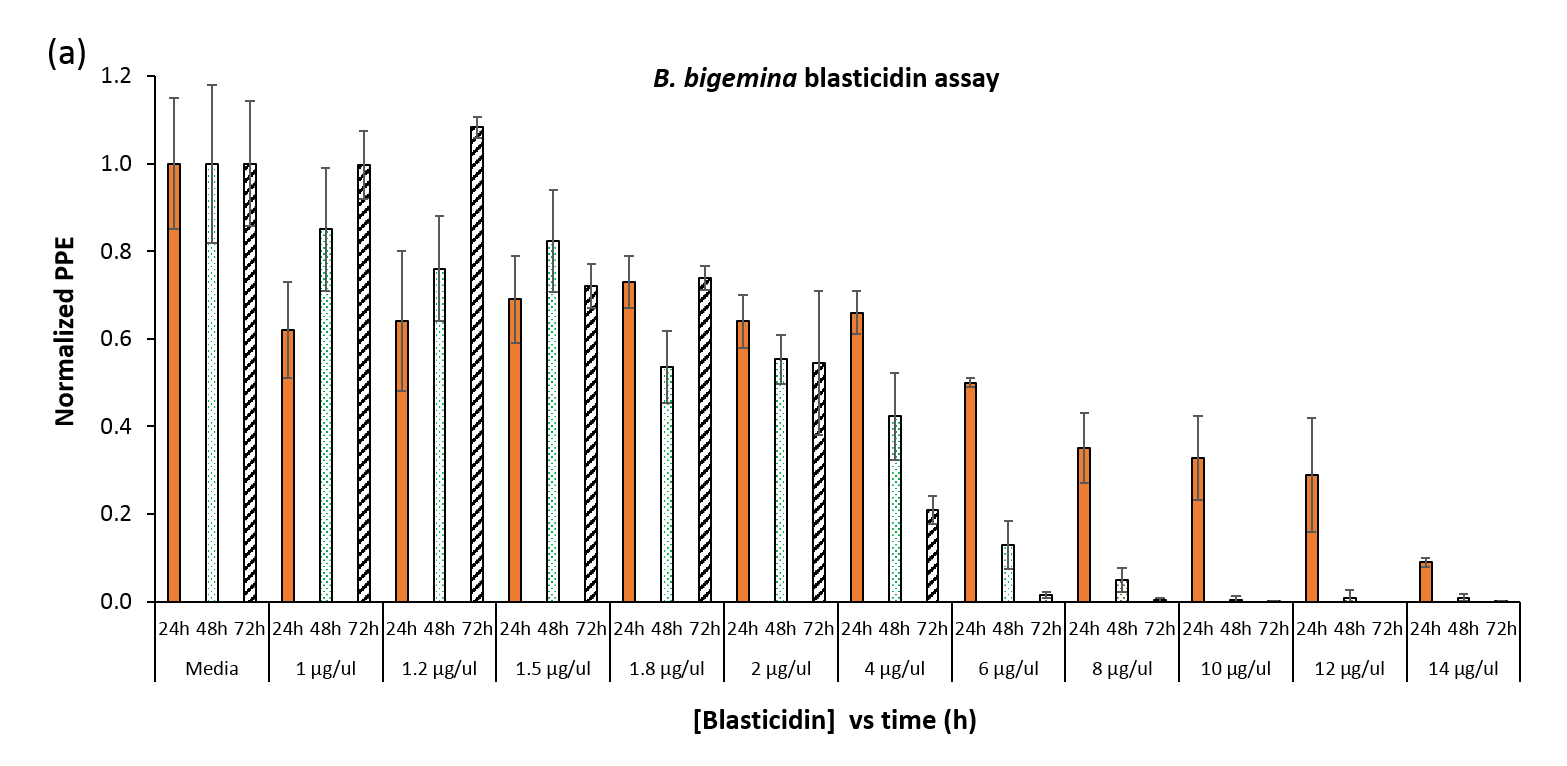
**

**
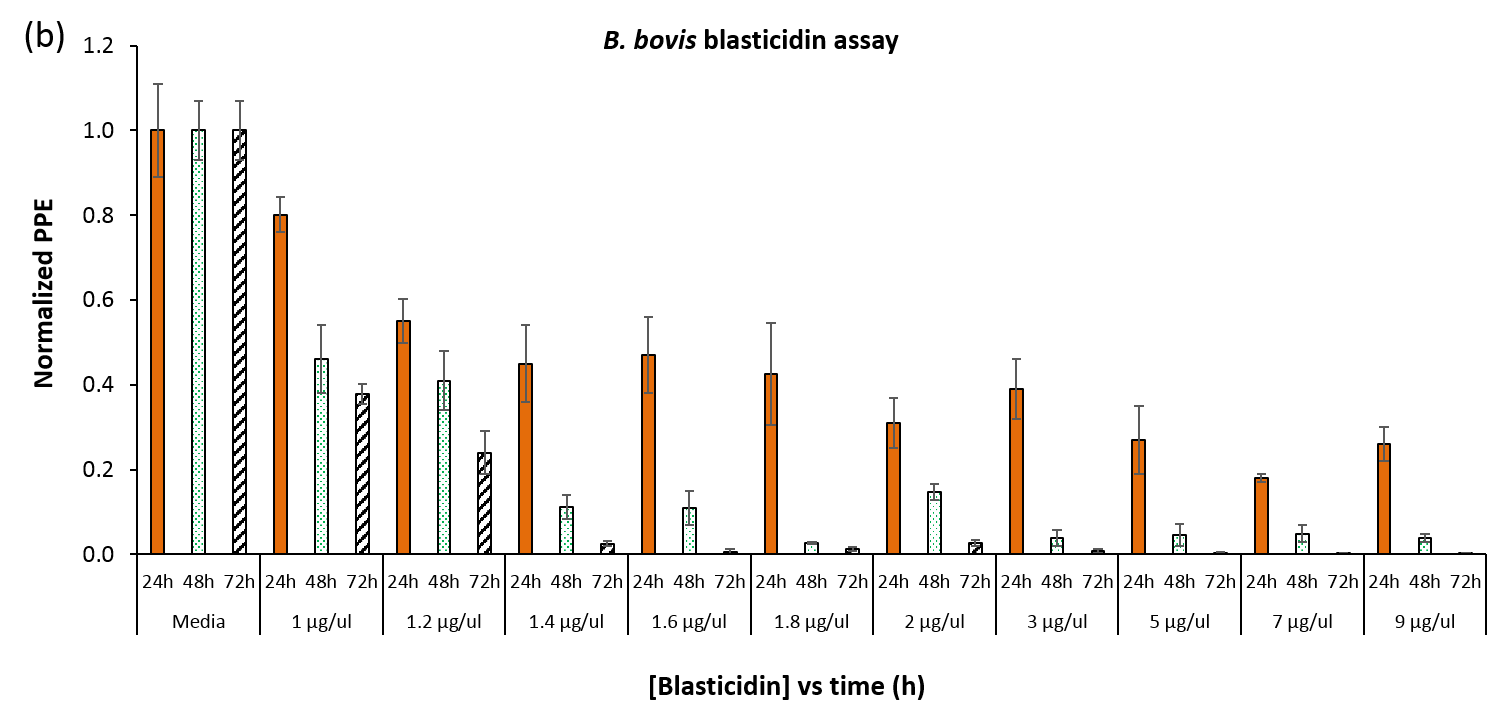
**

**Suppl. Figure 1.** In vitro growth curve of both non-transfected *B. bigemina* and *B. bovis* parasites in the presence of different concentrations blasticidin up to 72 hr. **(a)** *B. bigemina* growth curve. **(b)** *B. bovis* growth curve. Normalized PPE values (Y axis) obtained from *Babesia* spp. in the presence or absence of blasticidin (X axis). Media was used as negative controls for no inhibition. Error bars indicate standard deviations for each sample tested from triplicate culture.

**
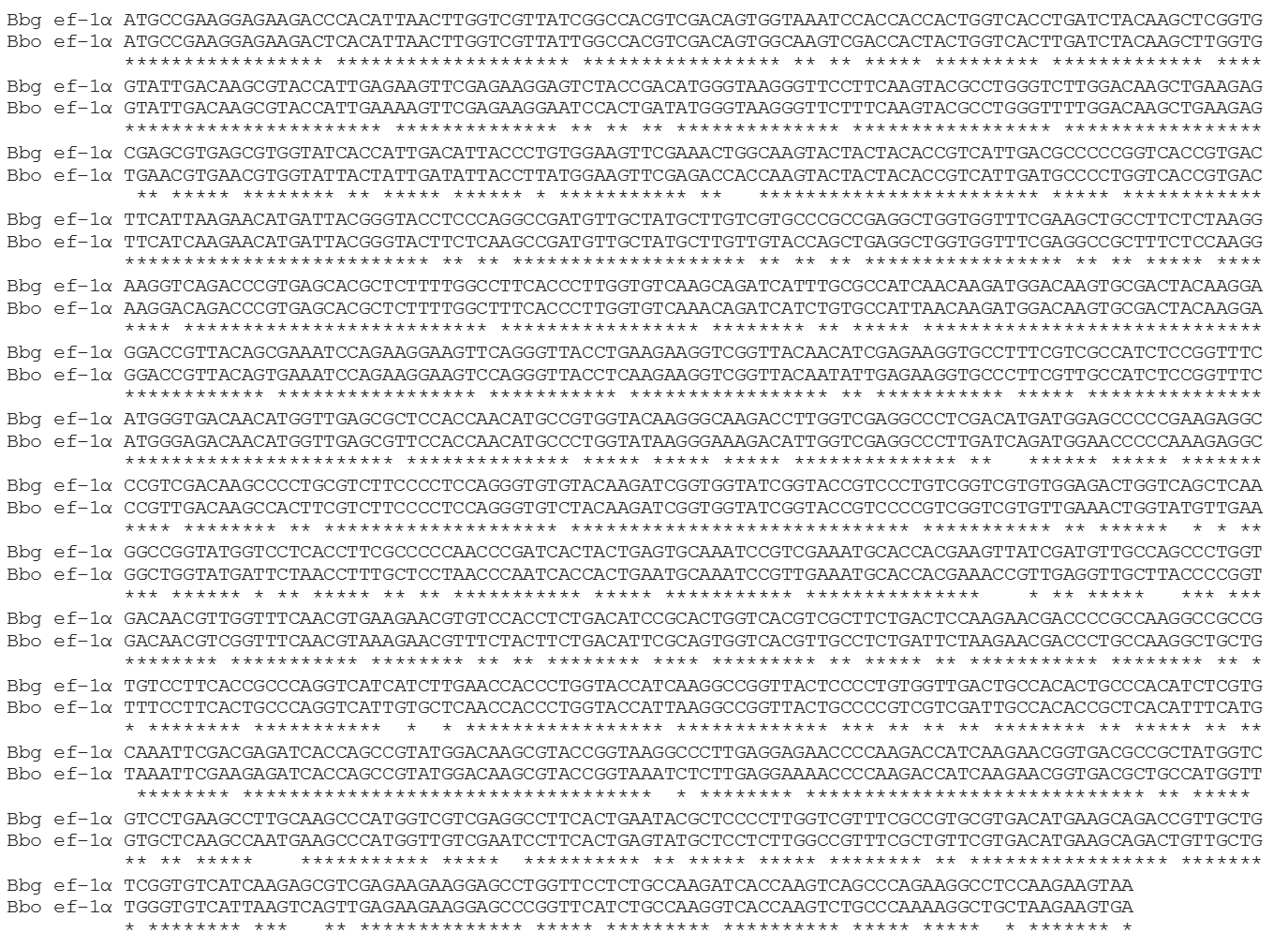
**

**Suppl. Figure 2.** Sequence comparisons among the ef-1α orf of *B. bovis* and *B. bigemina*.

**Suppl. Table 1.** Percentage of identity between *B. bigemina* *ef-1a* locus and *B. bovis ef-1α* locus

|  | *Bbig ef-1α* | *Bbo ef-1α* |
| --- | --- | --- |
| *Bbig ef-1α* | 100 | 87.45 |
| *Bbo ef-1α* | 87.45 | 100 |
